# Resource frontiers and agglomeration economies: The varied logics of transnational land-based investing in Southern and Eastern Africa

**DOI:** 10.1007/s13280-021-01682-z

**Published:** 2022-01-15

**Authors:** Dilini Abeygunawardane, Angela Kronenburg García, Zhanli Sun, Daniel Müller, Almeida Sitoe, Patrick Meyfroidt

**Affiliations:** 1 Earth and Life Institute, Georges Lemaître Centre for Earth and Climate Research (TECLIM), UCLouvain, 1348 Louvain-la-Neuve, Belgium; 2 Leibniz Institute of Agricultural Development in Transition Economies (IAMO), 06120 Halle (Saale), Germany; 3 Department of Forest Engineering, Universidade Eduardo Mondlane, 1102 Maputo, Mozambique; 4 Department of Historical and Geographic Sciences and the Ancient World, University of Padua, 35141 Padova, Italy; 5 Geography Department, Humboldt-Universität Zu Berlin, 10099 Berlin, Germany; 6 Integrative Research Institute On Transformations of Human-Environment Systems (IRI THESys), Humboldt-Universität Zu Berlin, 10099 Berlin, Germany; 7 FNRS, Fonds de La Recherche Scientifique, 1000 Brussels, Belgium

**Keywords:** Agricultural investments, Bayesian belief network, Decision model, Deforestation, Large-scale land acquisitions, Land use change

## Abstract

Actor-level data on large-scale commercial agriculture in Sub-Saharan Africa are scarce. The peculiar choice of transnational investing in African land has, therefore, been subject to conjecture. Addressing this gap, we reconstructed the underlying logics of investment location choices in a Bayesian network, using firm- and actor-level interview and spatial data from 37 transnational agriculture and forestry investments across 121 sites in Mozambique, Zambia, Tanzania, and Ethiopia. We distinguish four investment locations across gradients of *resource frontiers* and *agglomeration economies* to derive the preferred locations of different investors with varied skillsets and market reach (i.e., track record). In contrast to newcomers, investors with extensive track records are more likely to expand the land use frontier, but they are also likely to survive the high transaction costs of the pre-commercial frontier. We highlight key comparative advantages of Southern and Eastern African frontiers and map the most probable categories of investment locations.

**Supplementary information:**

The online version contains supplementary material available at 10.1007/s13280-021-01682-z.

## Introduction

For long, African frontiers in the dry woodland and savannah biomes have not received as much scholarly attention as their South American and Southeast Asian counterparts. The region was not known for high deforestation rates, state-sponsored land settlement schemes, and industrialized agriculture, those historical generalizations drawn from the trajectories of other frontiers (Rudel, 2013). However, the global land-based investments wave that started in the early 2000s and boomed later in the decade around the food and financial crisis have renewed the interest in African frontiers.

Despite a decline in new land-based investments in recent years (Land Matrix, 2019; UNCTAD, 2019), the effects of the existing investments on the social and political relations in agrarian systems have been profound (Borras Jr. et al., 2011; McMichael, 2012). The scale of land acquisitions, some involving thousands of hectares, their speculative nature (Cotula et al., 2009; Deininger et al., 2011; Fairhead et al., 2012), and numerous failures in consulting and compensating people (Vermeulen & Cotula, 2010; German et al., 2013) have caused suspicion over any potential benefits. While large-scale agricultural investments have the potential to generate economic and environmental gains through increased production (Deininger et al., 2011; Collier & Dercon, 2014) and land-efficient farming (Green et al., 2005; Balmford et al., 2018; Phalan et al., 2011), balancing these against social and environmental costs has remained a challenge. Reconciling these misaligned interests requires a broader understanding of not only the impacts of large-scale agricultural investments, but also the logics behind them.

Much has been written about the impacts of large-scale agricultural investments (Vermeulen & Cotula, 2010; German et al., 2013; Johansson et al., 2016; Ali et al., 2019; Davis et al., 2020; Liao et al., 2020), but an assessment of the logics behind these investments and how the logics and locations vary across heterogeneous investors have been missing. Our attempt here is to fill this major knowledge gap. Balancing agricultural gains against social costs and conservation priorities is critical for any rural development effort that encompasses land-based investments. The purpose of this work is to advance a policy dialogue around the types of investments that can best achieve this balance and the types of locations where this is most likely.

Despite the earlier efforts that were largely hampered by incomplete and inconsistent data (Deininger, 2011; Oya, 2013), recent research has made progress in characterizing different agricultural investment models and assessing their implications. These investment typologies consider organizational structures (e.g., ownership and funding), production models (e.g., types of production, scale, and value chain position), impacts (e.g., on labour, tenure, livelihoods, and the broader economy), and inclusiveness (e.g., in decision making, risk and benefit sharing, and access capital, inputs, and expertise) of the investments (Boche & Anseeuw, 2013; Hall et al., 2017 Chamberlain & Anseeuw, 2019; Giger et al., 2020). There have also been case studies that assess the distinct implications of different investment models on employment, people displacement, and economic growth (Hall et al., 2017).

Studies on investment location determinants stem largely from FDI (foreign direct investments) theory and the early works of location theory. The larger body of FDI literature focuses on country-level determinants in the industry and service sectors, and points to the usual suspects of market proximity, human capital (Noorbakhsh et al., 2001), bilateral ties, governance (Egger & Winner, 2005 Cuervo-Cazurra, 2006; Busse & Hefeker, 2007), and corporate taxes (Wei, 2000). The handful of studies that specifically focus on land-based investments find agroecological conditions, tenure security, land availability, investor protection, investor-host distance, colonial ties, and common languages to have an effect on location choices (Arezki et al., 2013; Lay & Nolte, 2017). However, these findings are mainly based on firm-level models that use country-level investment and covariate data (Blonigen, 2005). Qualitative analyses and interpretations have mostly relied on deductive normative frameworks, such as small versus large farms or family-owned versus capitalist operations (Oya, 2013). These limitations highlight the importance of a data-driven, inductive approach to better understand the logics of large-scale transnational agricultural investments in Africa.

Using an inductive grounded theory approach (Glaser & Strauss, 1967), we investigated who invests, where, and why, in the understudied frontiers of Southern and Eastern Africa. We specifically focused on transnational investors around whom the landgrab discourses are centred. Further, for African economies with scarce domestic capital, attracting foreign investments is critical for development. We combined firm- and actor-level interview and spatial data to (i) reconstruct the logics of investment decision making and (ii) identify the determinants of investment locations, given their heterogeneity across gradients of *resource frontiers* and *agglomeration economies, *using a Bayesian network (BN).

Our sample consists of 37 investments operated by 29 investors across 121 farm and plantation locations, covering 11% of the total transnational agricultural and forestry investments made between 2000 and 2016 in Mozambique, Zambia, Tanzania, and Ethiopia. According to the Land Matrix, between 2000 and 2016, these countries signed contracts for a total of 342 agriculture and forestry investments (see Table S1 for key development indicators of the four countries). Together the investments stretched over an area of 7.7 million hectares and amounted to 41% of the total number and 18% of the total area of land-based investments in Sub-Saharan Africa. In terms of the number of investments, around half the investments were intended for food production, 14% for biofuel production, 13% for non-food production, 9% for livestock, and 5% for forestry (Land Matrix, 2019). However, there were discrepancies between these figures and the actual number and the distribution of investments on the ground. For example, almost half of the failed investments comprised those intended for biofuel production (Nolte, 2020). While data on the types of investors for individual countries are unreliable, Africa-wide figures suggest that 43% are private investors, 16% are listed companies, and 12% are investment funds, with over fifth of the investors unaccounted for (Giger et al., 2020).

## Analytical Framework

Our framework of analysis builds on rent and FDI location theories. Classic rent theories of von Thünen and Ricardo model economic rent of the land as a function of the comparative advantage of market accessibility and bio-physical suitability, respectively. More recent works have focused on the endogeneity of firm interactions, producer prices, and the geographical distribution of demand in determining location choices (Krugman, 1998; Robalino & Pfaff, 2012; Garrett et al., 2013; Chamberlin et al., 2014; Richards et al., 2014). Beyond such comparative advantages, firm-specific characteristics also make a specific location attractive to certain types of investors. The *eclectic paradigm* of FDI theory refers to these firm-specific, spatially transferable, intangible assets that guide investment decisions as *ownership advantages—*which is separate from ownership of land (Dunning, 2001). Ownership (proprietary) advantages may consist of crop-specific expertise, technologies, managerial skills, and established brands. As economies integrate, such comparative advantages lead to external scale economies (Scotchmer & Thisse, 1992), local and regional specialization (Krugman, 1991; Ottaviano & Puga, 1998), and frontier expansion (Garrett et al., 2013; Richards et al., 2014).

**Fig. 1:**
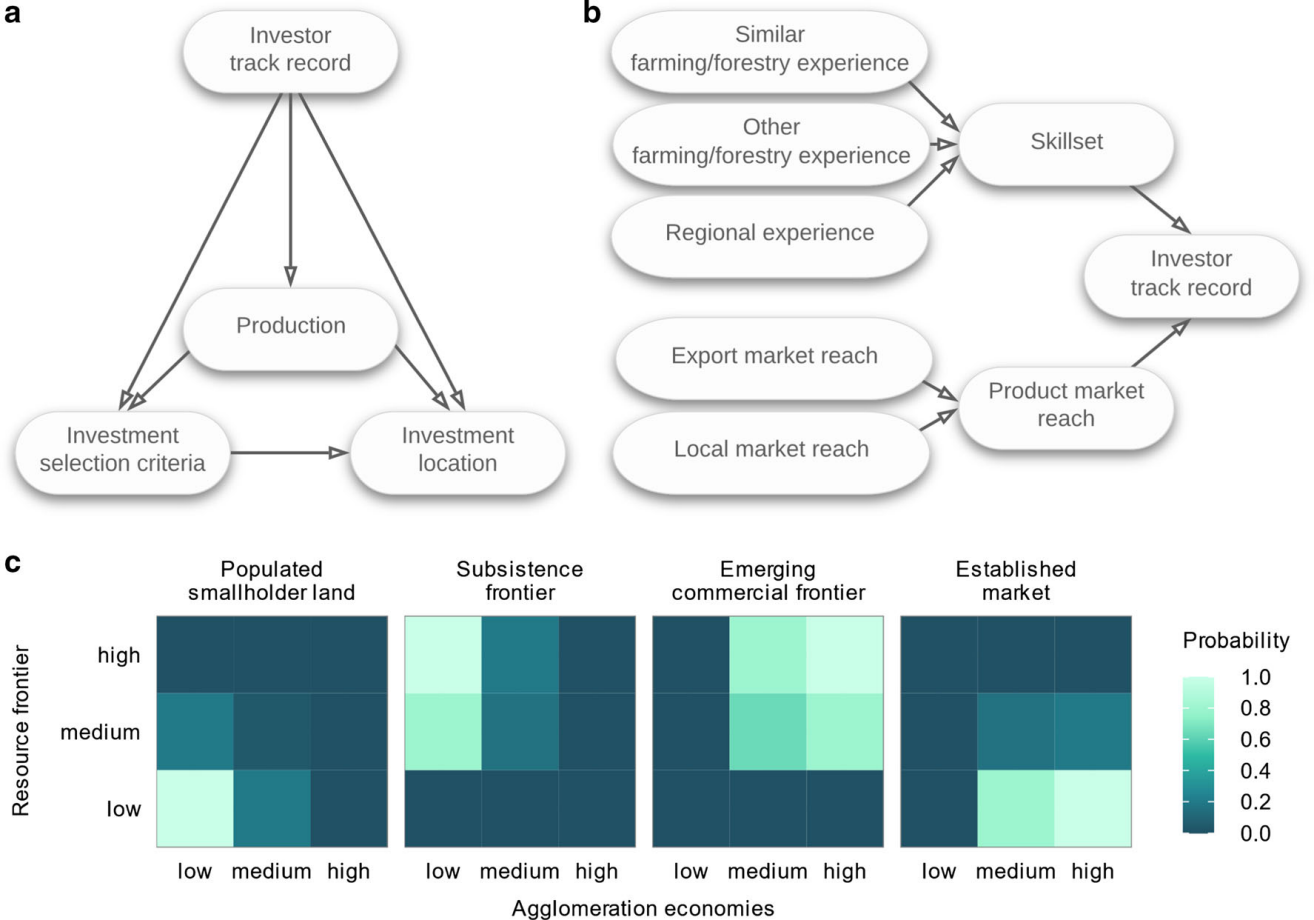
Conceptual model. **a** Interdependencies between the determinants of investment locations. It is hypothesized that the investment selection criteria enumerated by the investors, their production choices, and track records explain the choice of their investment locations. **b** Variables measuring investor track record. Investor track record is an aggregate measure of related skillsets and existing product market reach. Skillset is an aggregate measure of previous farming or forestry and regional experiences. Product market reach is an aggregate measure of existing export and local market reach (see Table S2). **c** A typology presenting the four investment location categories and their prior probabilities. An investment location is characterized by assessing it against an index combining the two variables *resource frontier* and *agglomeration economies*. An investment location is considered a *populated smallholder land*, if it is low on both *resource frontier* and *agglomeration economies* indices, a *subsistence frontier*, if it ranges medium to high on the *resource frontier* index and is low on the *agglomeration economies* index, an *emerging commercial frontier*, if it ranges medium to high on both *resource frontier* and *agglomeration* *economies* indices, and an established market, if it is low on the *resource frontier* index and high on the *agglomeration economies* index. The probabilities indicate the likelihood of each location belonging to one of the four investment location categories, and these probability scores are used as priors in parametrizing the BN (see Model under Methods and Tables S4–S6)

We hypothesize that a given investment location can be explained by a set of selection criteria enumerated by the investors, their production choices, and track record (Fig. 1a-b). We measured the appeal of investment locations to an investor in terms of *resource frontiers* and *agglomeration economies*. To operationalize these two measures, we drew upon the following definitions.

A *resource frontier* is delineated as a transformative moment between the discovery or the reinvention of an abundant resource and its consolidation (Barbier, 2012; Rasmussen & Lund, 2018). During this time, the resource, the means of its exploitation, and the institutional orders that govern them undergo reconfiguration, which is often marked by a dwindling resource base, accumulation of capital, and an increasing population (Barbier, 2012; Lambin et al., 2013; Meyfroidt et al., 2018; Rasmussen & Lund, 2018). Some key defining features of this reconfiguration amenable to modelling are the dwindling resource, in this case the land base, and the accruing labour force or capital inputs (Barbier, 2012; Meyfroidt et al., 2018).

We defined *agglomeration economies* as economies of scale external to the individual firm, but internal to the sector (Porter, 1996; Scott & Storper, 2003). Agglomerations create localized clusters of specialized knowledge, inputs, and industry-specific infrastructure and institutions. The process of clustering lowers transaction and production costs, promotes learning and innovation, increases local competition, and enables leveraging collective political agency (Krugman, 1991; Porter, 1996; Scott & Storper, 2003). As a result, agglomeration marks a key phase in commodity agriculture expansion (Garrett et al., 2013; Richards et al., 2014). Among these, features amenable to modelling *agglomeration economies* include, economic activity, market accessibility, the presence of large-scale agriculture, and sector-specific ancillary services.

*Resource frontier* and *agglomeration economies* encapsulate the compromise an investor makes in choosing cheap land with the potential to expand and achieve scale economies internal to the firm, as opposed to locating closer to an investment cluster to benefit from the existing scale economies that are external to the firm. Building on this premise, we derived a typology comprising four distinct investment location categories: (i) *populated smallholder land* which is low on both *resource frontier* and *agglomeration economies* indices, (ii) *subsistence frontier*, which ranges medium to high on the *resource frontier* index and is low on the *agglomeration economies* index, (iii) *emerging commercial frontier*, which ranges medium to high on both *resource frontier* and *agglomeration economies* indices, and (iv) *established market*, which is low on the *resource frontier* index and high on the *agglomeration economies* index (Fig. 1c).

## Materials and Methods

### Study sites

The four countries in our sample, i.e., Mozambique, Zambia, Tanzania, and Ethiopia are characterized by rural agrarian economies. A large fraction of the population is poor and derives its livelihood and incomes primarily from farming and related activities (Supplementary Information (SI), Table S1). The main crops grown in the region include cassava, maize, and sugar cane (FAOSTAT, 2019). The four countries have pluralistic land tenure systems which include different forms of customary or communal and statutory forms of land rights (UNECA, 2003). The state retains residual ‘ultimate ownership’, while private tenure is granted through rights of usufruct through lease entitlements that can range between 14 to 99 years (Crewett et al., 2008; German et al., 2013).

### The model

Building on rent and FDI theories and our interviews, we defined an investment location as a function of a set of selection criteria enumerated by the investors, their production choices, and track record (Fig. 1a). We defined investor track record as the aggregate measure of related skillsets, and existing product market reach of all the investors involved in a single investment (Fig. 1b). Skillset was measured in terms of previous farming or forestry and regional experiences, and product market reach in terms of existing local and export market reach (Table S2 presents how each of these aggregate variables was calculated). We operationalized these variables using interview and additional firm data (section Data below details the data used).

To quantify *resource frontiers,* we used the two spatial variables; (i) population density and (ii) the proportion of potentially suitable land yet to be converted to agriculture (Table S3 lists data sources). We combined these two measures into a single index, using elicited conditional probabilities (Table S4), which gauged each investment location along a *resource frontier* gradient. To quantify *agglomeration economies*, we used the two spatial variables; (i) economic activity and market accessibility and (ii) average field size, the latter to proxy the presence of large-scale, capitalized agriculture. Using elicited conditional probabilities, we calibrated a second index to combine these two measures (Table S5). We combined the two indices into a third to construct a typology comprising four distinct investment location categories, i.e., (i) *populated smallholder land* (ii) *subsistence frontier* (iii) *emerging commercial frontier* and (iv) *established market* (Table S6 and Fig. 1c).

### Data

Our efforts to establish contacts with investors were multiple and stretched over two years. We started with a list of transnational agricultural and forestry investments in Mozambique, Zambia, Tanzania, and Ethiopia extracted from the Land Matrix (Land Matrix, 2019). In choosing the sample countries, we focused on investor hotspots within Southern and Eastern Africa that were common targets, with adjustments to capture the heterogeneity of the investment contexts. We first reached out to the management of some companies, using contact details made available on the company website or through personal contacts. We then adopted a snowball sampling method, relying on investors with whom we had already established contact to introduce us to other investors. Some of the investments that were listed in the Land Matrix did not exist on the ground (e.g., biofuel investments) and some investments were not included in Land Matrix (e.g., out-grower schemes). There were other investments that operated under different names to those that were listed and yet others that had already been transferred to other investors. Prior to the interviews, we shared the study objectives, data expectations, intended analyses, and the ways in which the data will be used, with the participants by email or phone. We guaranteed anonymity to all the participants. Upon request, we entered into non-disclosure agreements with some participants. Some investors who declined to participate or refrained from responding at the beginning did take part in the study toward the end. The final sample included 37 investments operated by 29 investors across 121 farm and plantation locations.

To generate actor- and firm-level investor data, we carried out 62 semi-structured interviews across the management chain. The top-level management, including chief investment officers (CIOs), chief executive officers (CEOs), managing directors, and investors, provided information on land use decisions such as production choices (e.g., crops, livestock, horticulture, or forestry species) and locations. The farm and country managers filled in with details on day-to-day management and operations. We conducted 32 additional interviews with local investors and other actors from international organizations, civil society, international and national NGOs, state departments, and academia to better understand the prevailing narratives and the larger investment context. Data were collected using a semi-structured questionnaire, which was developed by building on previous works and information gathered during a pilot field visit (see SI for the questionnaire). All the interviews were carried out on site, except for four virtual interviews. Each interview lasted approximately 1.5–2.5 h and served to collect data on investment locations, the selection criteria adopted in choosing the investment locations, cultivated crops, and the company profiles including previous farming experience, regional experience, and product market reach. The interview data was complemented with additional information on the investments extracted from company annual reports, company websites, online news reports, and literature.

To generate the spatial data, we initially geolocalized 268 farm and plantation sites managed by the investors we sampled, using field records, Google Earth, and secondary spatial administrative data (e.g., land use title data for plantations and forestry concessions in Mozambique). We applied a 30 km buffer, to eliminate the neighbouring farms that were a part of the same investment, which brought the number of spatially distinct farms and plantation sites down to a total of 121. We used the distance between farms managed under a single investment and the farm sizes as guides to set the buffer size. Farms managed by a single investment were often within a 7–8 km radius, but the furthest were located at around a 30 km radius. The large farms in the region ranged between 2,000 and 3,000 ha (i.e., 20–30 km^2^). Due to their vast expanses, forest plantations managed by a single investment spread over much larger ranges. We therefore chose the upper limit of the distance between farms (i.e., 30 km) as the buffer size to extract variables characterizing investment locations. This permitted a trade-off between the spatial dependence in the data and the context beyond the farm and plantation boundaries. Then, we compiled a database of spatial variables with land cover, population density, field size, and market influence (Table S3). For each farm or plantation location, we calculated the mean value of population density, field size, market influence, and the proportion of each land cover class within a 30 km buffer using ArcGIS™. A link to anonymised data is provided in SI.

### Data limitations and selection biases

We have produced a unique data set on investment logics, based on extended hours of interviews with over 60 managers including top-level decision makers from a wide geographical area across Southern and Eastern Africa. These investors are often deemed unapproachable both at the individual and firm level. To the best of our knowledge this study is a first of its kind that relies on extensive interview data from a larger sample of top-level investors.

The sample is subject to potential selection biases. The investors who took part in the study did so on a voluntary basis. Since certain types of investors can be more open to participation than others, voluntary participation could bias the sample. We cannot rule out the risk of selective information sharing, either. However, given our approach to sampling, i.e., two years of networking with investors, snowball sampling, an anonymity assurance, extended interview hours, and multiple interviews across the management chain from a single investment, we assume this risk to be minimal. Although there are many inconsistencies and biases associated with the Land Matrix (Nolte et al., 2016), we only used the database to compile an initial list of companies and contacts. As we progressed, we revised and adapted this list to the ground situation. Therefore, we do not believe the biases associated with the Land Matrix to have any considerable effect on our sample.

It is important to note that the pool of transnational land investors in Africa is small, which is reflected in our sample that contains 39 different investments but only 29 individual companies. For these reasons, we believe that our sample is reasonably representative.

### Bayesian network (BN)

We converted the conceptual framework (Fig. 1) into a directed acyclic graph (DAG), as shown in Fig. 3 and operationalized it in a BN using firm- and actor-level interview and spatial data (see Variables in SI).

A BN is a probabilistic graphical model which encodes the joint probability distribution of a set of random variables. It comprises two components: (i) a qualitative component, which is the DAG that represent the interdependencies between variables (i.e., nodes), and (ii) a quantitative component with conditional probability tables (CPT) that quantify the strength of the conditional dependencies between the variables using a set of parameters (Jensen & Nielsen, 2007; Pearl, 2009). Each node in the DAG denotes a variable which is an attribute, feature, or hypothesis about an uncertain event with a set of state values. These state values are mutually exclusive, collectively exhaustive, and typically discrete. The directed arrows pointing from parent (i.e., predictor) to child (i.e., target) nodes capture cause-effect relationships and/or statistical correlations (Aguilera et al., 2011). The CPTs quantify the strength of the influence of the predictor variables on the target variables, given the combination of the discrete states of all the predictor variables. Mathematically, the conditional interdependencies among the variables are derived using Bayes’ theorem (Heckerman et al., 1995), which links the probability of hypothesis H conditional on evidence E as $P\left(H|E\right)=P\left(E|H\right)\times P\left(H\right)/P\left(E\right)$. The joint probability distribution over a set of variables ${X}_{1}$, ${X}_{2}$, … ${X}_{n}$ in a BN can be factorized as $P\left(X\right)=P\left({X}_{1},{X}_{2},{\dots X}_{n}\right)={\prod }_{i=1}^{n}P\left({X}_{i}|{X}_{pa\left(i\right)}\right)$, where ${X}_{pa\left(i\right)}$ indicates the set of parent variables of ${X}_{i}$ (Kjærulff & Madsen, 2013).

BNs can incorporate multiple levels of randomness to combine qualitative and quantitative data and capture the uncertainty and variation inherent in observation and parameter estimation (Hackerman et al., 1995; Gelman et al., 2003; Sun & Müller, 2013). These attributes were particularly amenable to modelling the uncertainties inherent in decision making, given that we worked with a small interview dataset with some missing values. Our BN comprised 14 predictor variables including two investor profiles and 12 variables that capture investment selection criteria. The two outcome indices, i.e., *resource frontier* (Table S4) and *agglomeration economies* (Table S5), were each calibrated using two spatial variables. A final output typology, i.e., investment location was derived by combining the two outcome indices (Table S6). Table S2 details the individual variables (BN nodes), their states and thresholds, and the observed variables used in calculating CPTs. We used Netica™ Version 5.24 software to implement the BN, which includes a graphical interface and built-in algorithms for Bayesian inference and imported the outputs and sensitivity scores to R for further analysis and plotting (Wickham, 2016; R Core Team, 2020). A link to R code is provided in SI. We validated the model by conducting a validation exercise with a selected group of investors and researchers (see Model validation in SI).

To assess the sensitivity of a variable with respect to another, we used entropy reduction metrics. This measure, also known as mutual information, calculates how much knowing one variable reduces the uncertainty of knowing the other (Pearl, 1988; Marcot, 2012).

### Maps

We combined four spatial datasets, i.e., land cover, population density, field size, and market influence, and derived an investment location map using Bayesian parameter estimation. Different land cover classes occurring in the region were reclassified as *unconverted land*. Finer resolution data was resampled at the coarsest resolution (Table S3). For each grid cell, we extracted the raster values of land cover, population density, field size, and market influence and calculated the proportion of unconverted land. We processed the four spatial variables in Netica™ to generate the probability of each grid cell belonging to any of the investment location categories, i.e., *populated smallholder land*, *subsistence frontier*, *emerging commercial frontier*, and *established market*.

We extracted the most probable investment location category of each grid cell to map the types of investment locations (Fig. 4a). Using the probability of each grid cell belonging to any of the four investment location categories, we calculated the Shannon index (H) (Shannon & Weaver, 1949) of each cell to map the uncertainty of investment locations (Fig. 4b). H quantifies uncertainty or entropy based on the weighted geometric mean of the proportional abundance of the type of elements in a set, using $H=-\sum_{i}^{n}{p}_{i}\mathrm{ln}{p}_{i}$, where *i* is a type of element in a set of *n* types of elements and *p* is the proportion of the *i*th element type. We mapped the Shannon index scores into three discretized low-medium–high categories using equal intervals. We masked the arid zones with less than 60 days of growing period (IIASA/FAO, 2012) and the IUCN-designated protected areas of the categories I through VI (Protected Planet/ IUCN/UNEP, 2017), considering that these regions are in principle void of investments. See SI for a link to spatial data and code.

The investment locations characterize the variability in *resource frontiers* and *agglomeration economies* and does not correspond to a suitability analysis.

## Results

### The investors, investments, and key selection criteria

Our sample contained three main types of investors—*agribusinesses* (55%), *fund managers* (28%), and *development finance institutions* (DFIs; 17%). These investors originated from the UK, South Africa, India, Singapore, USA, Saudi Arabia, Norway, and the Netherlands. We identified *agribusinesses* as those investors who create value through production and *fund managers* as those who generate value through rentier relations, i.e., extracting value through financial transactions involving commodities or property rights rather than through production (Meyfroidt et al., 2019). We identified those who invested with an underlying goal to create jobs and induce scale economies, in places where these would otherwise be unlikely, as *DFIs*. Individual investors (i.e., an individual or a single business entity) in our sample largely corresponded to these three predominant investor types, which have been identified also by previous works (Daniel, 2012; Boche & Anseeuw, 2013; Ouma, 2015; Ducastel & Anseeuw, 2017; Kish & Fairbairn, 2017). However, the actual investments were often made up of several investors forming new configurations that constantly adapted to the prevailing constraints. Such configurations included corporate agribusinesses who managed supply chain logistics and sold in partnership with small farmers; fund managers who leased out land to contract farmers; and medium- to large-scale agriculture ‘projects’ in which all three types of investors had invested. These investments did not represent the profile of a single partner, but rather an amalgam of the individual investors’ skillsets, experiences, market reach, and motives. Therefore, unless otherwise specified, we report our findings by the investment and not by the investor.

In terms of production, we separated the operations (*n* = 121) into three main types, i.e., *high-value food crops* (11%), including coffee, macadamia nuts, and deciduous fruits such as apples, avocados, citrus, litchi, and pears, *other agriculture* (30%), including banana, corn, cotton, maize, peas, potato, soya, tobacco, vegetables, horticulture, and livestock, and *forestry* (59%), including eucalyptus and pine species.

**Fig. 2:**
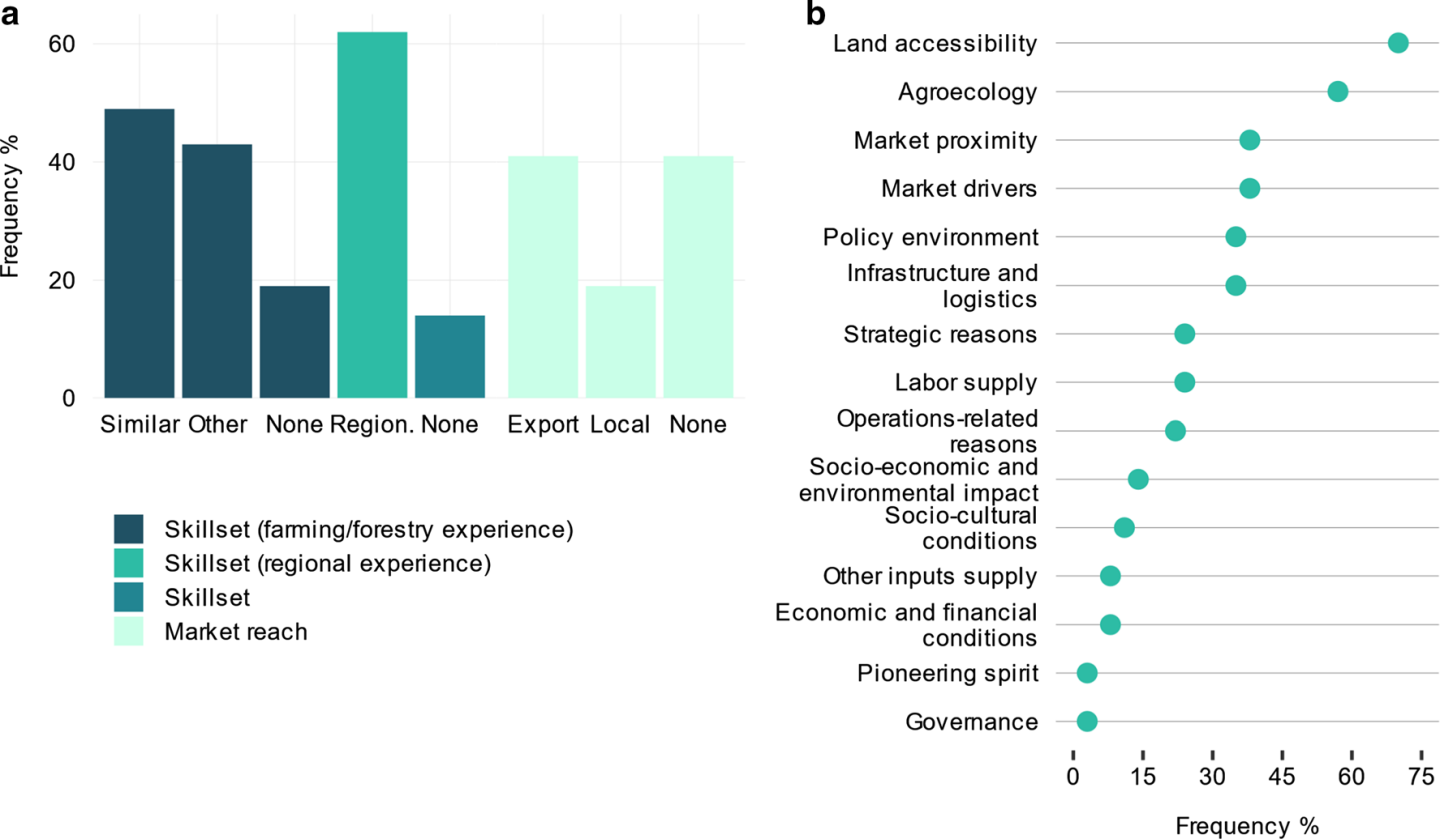
Investor sample characteristics. **a** Investor track records including skillsets and market reach (n = 37). The columns, in the order from left to right, show the frequency of investors: with previous farming or forestry experience in the exact same or similar production as the target production of the investment under study (Similar); with farming or forestry experience in other production (Other); without farming or forestry experience (None); with regional experience (Region.); without sector-related (i.e., farming or forestry and regional) skillsets (None); with existing export market reach for the exact same or similar production as the target production (Export); with existing local market reach for the exact same or similar production (Local); and without export or local market reach for the exact same or similar production (None). **b** The frequency of the key groups of investment selection criteria enumerated by the investors (n = 37). See Table S2 for a disaggregation of the individual variables cited by the investors that make up the key groups of selection criteria summarized here

In terms of investor *track records* (Fig. 1b and Table S2), i.e., the aggregate effect of skillsets and product market reach, 38% of the investments (*n* = 37) were made up of investors that had an *extensive track record* (Fig. 2a). Almost half of the investments (49%) recorded previous farming or forestry experience similar to the production types targeted by the current investment. On the contrary, 19% lacked any farming or forestry experience. Around 62% of the investments had previous commercial experience in the region and 14% had no related skillsets. In terms of product market reach, 41% of the investments had an established export market for the target crop and 19% an established local market. Around 41% of the investments were new to product markets.

The investors identified over 54 criteria that had guided and informed their choice of investment locations and production types (Table S2). We aggregated these into key groups of selection criteria (Fig. 2b). The top six most frequently cited groups of criteria in descending importance were, land accessibility, agroecology, market proximity, market drivers, infrastructure and logistics, and policy environment. Contrary to widespread narratives, governance conditions (defined by political stability, bureaucracy and red tape, and rule of law) were not of high priority in guiding investment decisions within Southern and Eastern Africa. This is consistent with global-scale studies using secondary data that found general institutional quality including corruption to have an insignificant effect on land-based investments (Arezki et al., 2013; Lay & Nolte, 2017).

### Investments Focus on Pre-Commercial Agriculture Frontier Areas

**Fig. 3:**
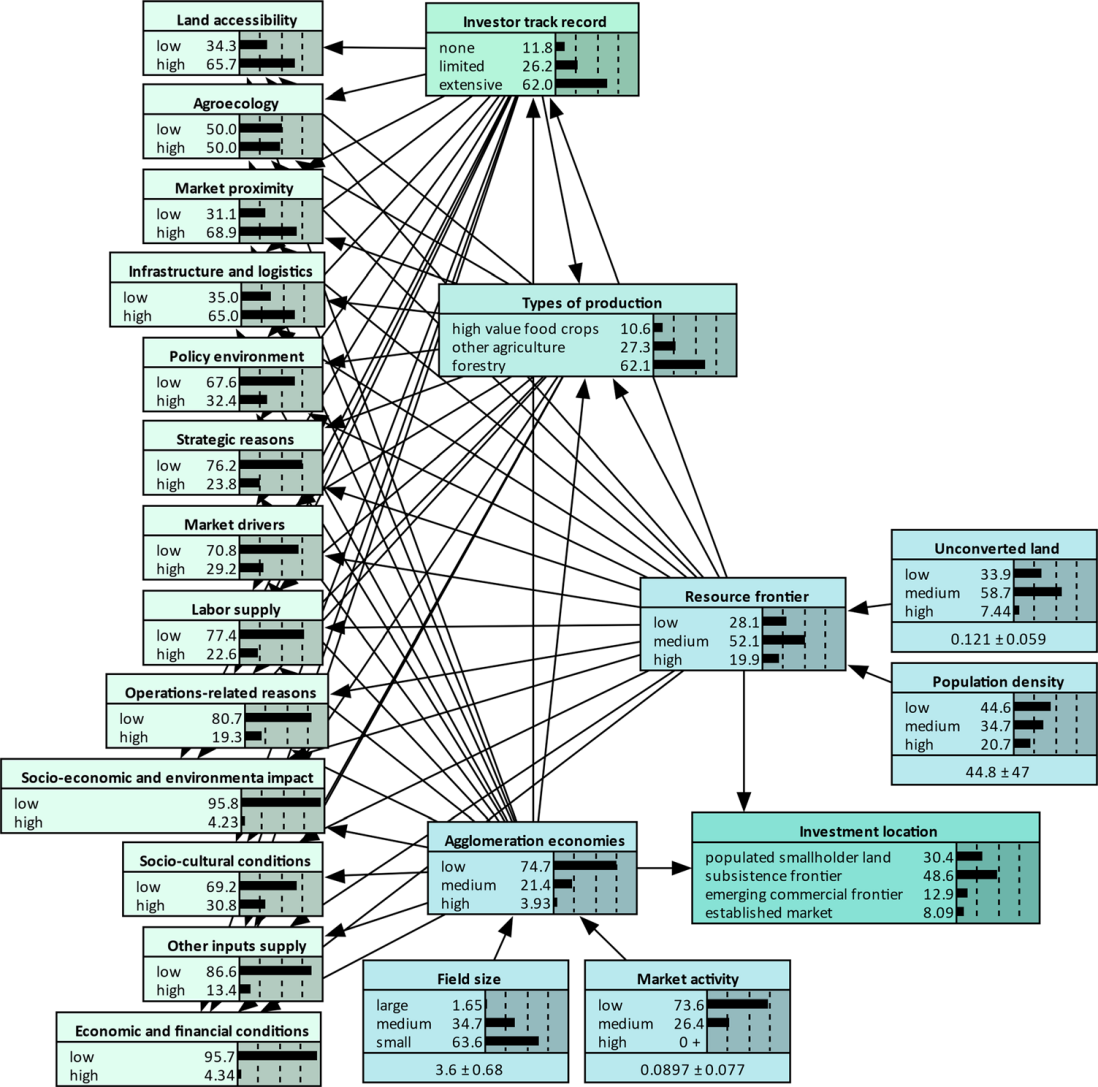
The Bayesian network (BN). The DAG (directed acyclic graph) represent the interdependencies between the determinants of the investment locations in the African frontiers. The results present the unconditioned determinants of a random investment location. The BN was parametrized first using the prior probabilities for the *agglomeration economies*, *resource frontier*, and investment location nodes (see Table S4–S6); and second using spatial data (n = 121) for field size, market activity, unconverted land, and population density; investment selection criteria enumerated by the investors (n = 37); types of production (n = 37); and investor track records (n = 37). See Bayesian network (BN) under Methods

BN results show the probability of an investment location, given investor track record, types of production, and investment selection criteria (Fig. 3). Based on these probabilities, over half of the investments (52%) occur in regions where *resource frontier* conditions are moderate and around 20% in regions where *resource frontier* conditions are high. Three quarters of the investments occur in regions with low *agglomeration economies*. From among the four investment location categories we derived based on the trade-offs between *resource frontier* conditions and *agglomeration economies*, *subsistence frontier* register close to 49% of the investments. *Populated smallholder land* register around 30% of investments, and *emerging commercial frontier* 13%.

**Fig. 4:**
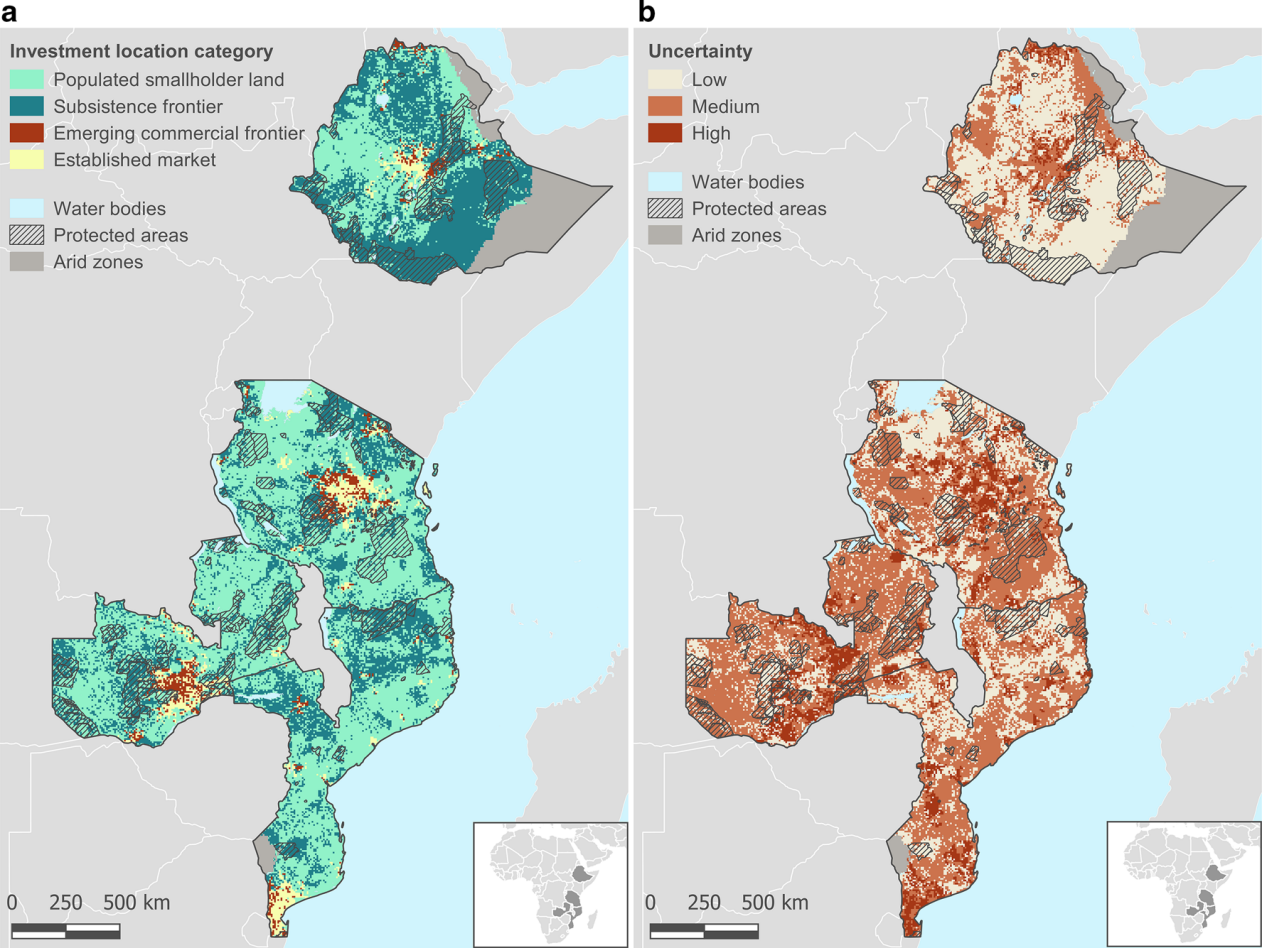
Types of investment locations across the four sample countries. **a** The most probable investment location category. The map shows the most probable investment location category assigned to each grid cell, out of the four investment locations, i.e., *populated smallholder land*, *subsistence frontier*, *emerging commercial frontier*, and *established market*. The probability of each grid cell belonging to one of the four investment location categories was calculated using the BN (see Maps under Methods). Based on area calculations, 55% of the land is populated smallholder land, 37% is subsistence frontier, 5% is emerging commercial frontier, and 3% is established market. **b** The degree of uncertainty is associated with the probability of investment location. The map shows the uncertainty of each grid cell belonging to a single investment location, calculated using the Shannon Index. The uncertainty in assigning a grid cell to an investment location category is low in 37% of the land, medium in 51%, and high in 12%. Protected areas are the IUCN-designated categories I through VI. Arid zones are the FAO/GAEZ designate areas reporting less than 60 days growing period

Using the BN, we also assessed the probability of any given location (grid cell) belonging to one of the four investment location categories (Fig. 4). Based on these probabilities, around 55% of the land across the four countries is *populated smallholder land*, 37% is *subsistence frontier*, and only less than 5% *is emerging commercial frontier*. While most of the land is in subsistence or semi-subsistence state, almost half of the investments occur in frontiers, where land resources are in relative abundance, but scale economies external to individual firms are lacking. Therefore, we find that agricultural frontiers in Southern and Eastern Africa are predominantly in a ‘precommercial’ state.

### Investment locations depend on investment selection criteria, investor track records, and types of production

**Fig. 5:**
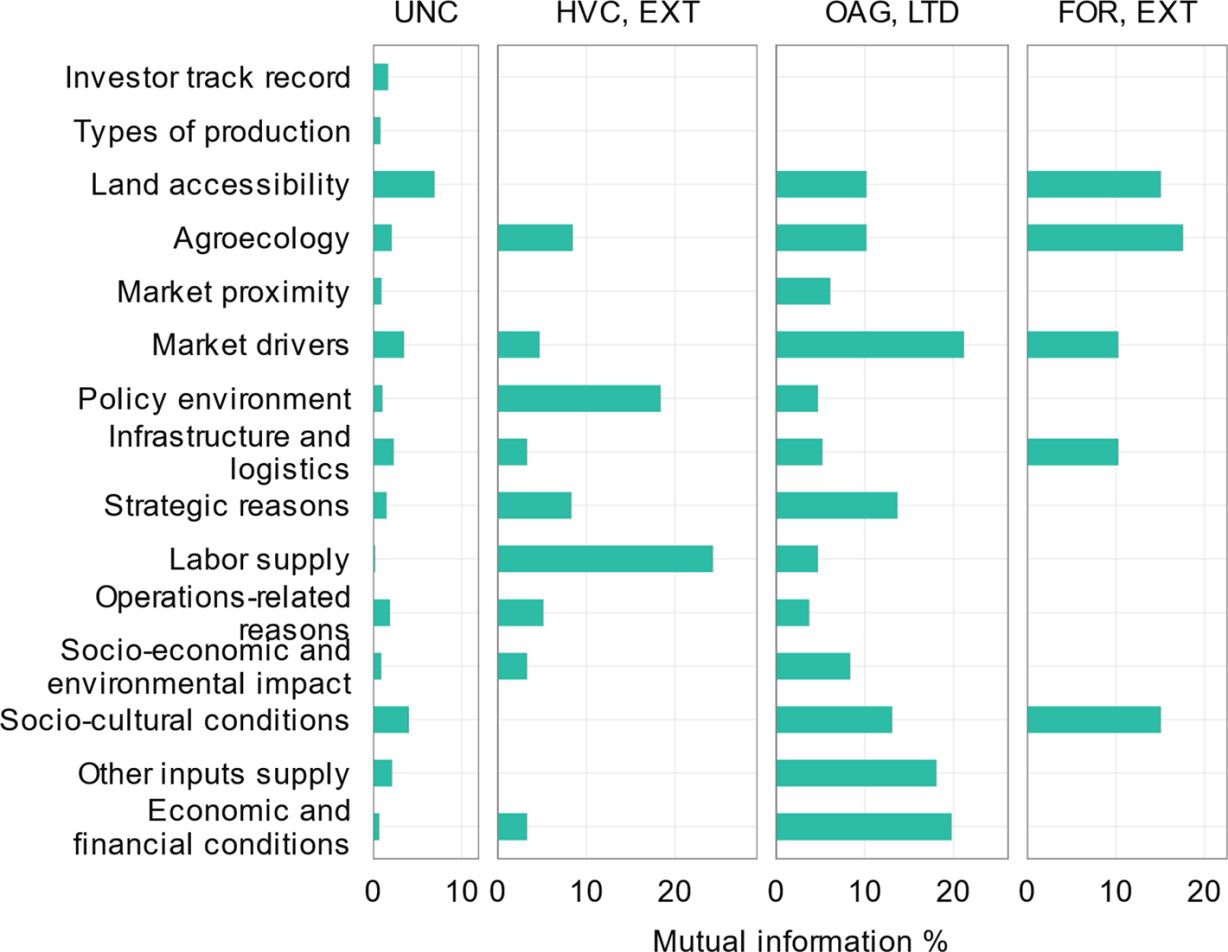
The sensitivity of BN variables. The sensitivity of variables measured in terms of mutual information quantifies how much knowing one variable reduces the uncertainty of the other. The panels show the sensitivity of each variable to the investment location corresponding to the unconditioned determinants (UNC) and when conditioned on investor track records and types of production as in high-value food crop investors with an extensive track record (HVC, EXT), other agriculture investors with a limited track record (OAG, LTD), and forestry investors with an extensive track record (FOR, EXT)

Our results show that an investment location is more sensitive to investment selection criteria and investor track records than to production types. Among selection criteria, sensitivity of an investment location is highest to land accessibility, socio-cultural conditions, and market drivers (Fig. 5).

**Fig. 6:**
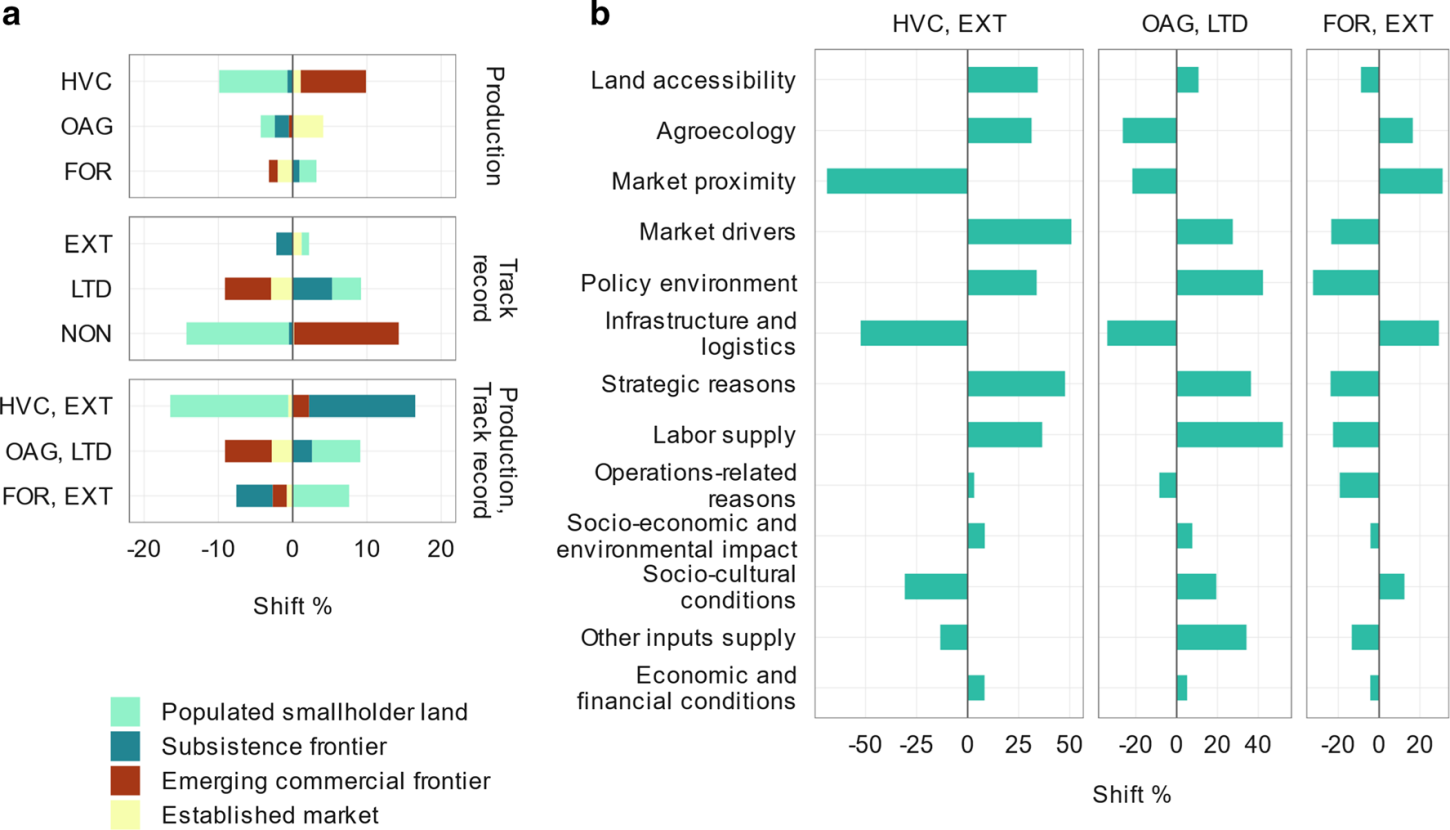
Shifts in the likelihood of investment locations and investment selection criteria. **a** Shifts in the likelihood of investment locations conditional on the following: different production types including high-value food crops (HVC), other agriculture (OAG), forestry (FOR); investor track records including extensive (EXT), limited (LTD), and none (NON); and production types and track records including high-value food crop investors with an extensive track record (HVC, EXT), other agriculture investors with a limited track record (OAG, LTD), and forestry investors with an extensive track record (FOR, EXT). **b** Shifts in the importance of investment selection criteria, when conditioned on investor track records and types of production as in high-value food crop investors with an extensive track record (HVC, EXT), other agriculture investors with a limited track record (OAG, LTD), and forestry investors with an extensive track record (FOR, EXT). These results are based on the BN

Given that almost half the investments occur in *subsistence frontier* (Fig. 3), it remained the most likely investment destination for most interactions between production types and investor track records. However, conditioning on different interactions resulted in considerable shifts in the relative choices of investment locations (Fig. 6a) and selection criteria (Fig. 6b).

When conditioned on production types, investments into high-value food crops favour *emerging commercial frontier*, while investments into other agriculture favour *established market* (Fig. 6a).

When conditioned on track records, investments that register extensive track records rely on fewer selection criteria, in contrast to those with limited track records, who rely on the entire range of selection criteria to make their investment choices (Fig. 5).

In terms of interactions between production types and track records, those with extensive track records investing in high-value food crops strongly favour *subsistence frontier* (Fig. 6a). The investment criteria they prioritize include market drivers, strategic reasons, labour supply, policy environment, land accessibility, and agroecology (Fig. 6b). In contrast, forestry investors with an extensive track record favour *populated smallholder land* (Fig. 6a). They weigh more on market proximity, infrastructure and logistics, agroecology, and socio-cultural conditions (Fig. 6b). Investors who invested in other agriculture with a limited track record favoured *populated smallholder land* and to a lesser extent *subsistence frontier* (Fig. 6a). These investors prioritize labour supply, policy environment, other inputs supply, strategic reasons, and socio-cultural conditions (Fig. 6b). The conditional shifts in the selection criteria prioritized by the investors (Fig. 6b) are further confirmed by the sensitivity measures (Fig. 5).

### Comparative advantages in southern and eastern african frontiers

Here, we combine our quantitative findings with the qualitative narratives extracted from the interviews to identify four comparative advantages that investors seek in Southern and Eastern Africa. These include: (i) large tracts of agroecologically suitable land, (ii) agroecological niches, (iii) potential market access, and (iv) location advantages conditional on investor track records. We found the investors’ response to these comparative advantages to be heterogeneous.

i. Large tracts of agroecologically suitable land
  Land has been and still is the main comparative advantage for investing in Southern and Eastern Africa. It offers vast tracts of land with low population densities, adequate year-round irrigation potential, and increasingly, brownfields (i.e., already established farms or plantations available for investing). The extent, level of occupancy, and water resources are known determinants that have attracted investments to the region (Burger et al., 2012; Arezki et al., 2013; Johansson et al., 2016; Lay & Nolte, 2017). However, the increasing number of brownfield investment opportunities is a relatively new phenomenon. Despite the ‘availability’ of land, large-scale land-based investments in Africa have suffered from tenurial conflicts and high farm establishment costs. Brownfields present solutions to these major obstacles in the form of existing title deeds and farm infrastructures or established plantations. The importance of brownfields in defining African frontiers may be an effect of the setbacks and failures experienced by the investments made in the early 2000s.
ii. Agroecological niches
  The pursuit of land that offers unique agroecological conditions that certain crops demand also attracts investments to Southern and Eastern Africa. Our results show that investors who focus on high-value crops with specific agroecological requirements are more likely to move deep into scarcely populated frontiers in pursuit of such land. For example, an investment into macadamia nuts had prioritized land that offered the unique growing conditions but limited market access, over land with generic agroecological conditions but excellent market access. An investor who supplied avocados to international markets and required guaranteeing year-round supply had sought land with a climate that provided a suitable crop calendar to fill gaps in existing supply. An investor growing seed potato had looked for land that was spatially isolated from other potato farms to minimize the risk of pest infections.
iii. Potential market access
  Market potential was another attraction, although much of it was driven by anticipation. Among those investors, whose investment decisions were guided by product markets and the availability of infrastructures and logistics, some responded to a speculative local demand signalled by a growing African population. Some responded to a speculative export market that was expected to benefit from the growing affluence in Asian markets. Investments that took place through state or donor-sponsored investment schemes in particular (e.g., Nacala and Beira corridors in Mozambique, the Southern Agricultural Growth Corridor of Tanzania known as SAGCOT, and the Malonda Foundation in Mozambique) relied on infrastructure development and economic incentives that were promised but hitherto had rarely been realized.
iv. Location advantages conditional on investor track records

So far, we presented advantages that are characteristic of the location and supposedly offer opportunities to all investors alike. Yet, there are location determinants that are specifically advantageous to certain investors owing to their firm-specific ownership advantages. Such location advantages conditional on crop-specific knowledge, technologies, managerial skills, or established brands are unique to the investor. For example, an investor who had established supply chains, gained managerial proficiency, and amassed information networks in Mozambique, continued to invest in the country, regardless of superior location advantages in a different country. Investors with extensive track records are also likely to pursue unique location advantages tied to the crops they specialize in. For instance, coffee in the Harar region in Ethiopia offers geographic reputation. Given the high transaction costs of operation, only certain established brands could adequately exploit this advantage. Growing tobacco in the Tete province in Mozambique benefited from a large pool of customary labour specialized in the crop, with whom an investor had a long-term trust relation. Driven by a vision to further strengthen core competencies and remain competitive, such investors invest in the pre-commercial frontiers, despite the less-than-ideal market conditions and infrastructures.

## Discussion and conclusion

Depending on varied investment logics, different investors value and prioritize different aspects of the land. Some investors invest in Southern and Eastern Africa because it is the ‘last frontier’. Some invest in the region because it is where the right growing conditions are. Others invest because a market awaits, and yet others venture new business opportunities to diversify money making or pioneer new land to make a difference. In practice, these intentions are not mutually exclusive and can work in tandem:

“We approach our investments from a geographic standpoint, and it marks the convergence of three things. First, it is the technology, it is unlocking natural assets that weren’t accessible before. Second, investing in natural capital that also contributes toward rural development presents a strong investment thesis, especially in Africa, because it is the last frontier for forestry and agriculture; and third, investments that were historically limited to the investment community is now collaborating with the development community. They are mixing the capital. What it does is, it de-risks the investment proposition.”

(A CEO of a management fund).

Investors’ interest in African land is not new (Cotula et al., 2009; Deininger et al., 2011). Yet, formerly unexamined nuances and complexities exist and understanding these are critical for the sector’s development. As the investor we quoted above eloquently articulates, capital markets and investment intelligence that evolve alongside technological advancements will continue to make the ‘last frontier’ desirable and accessible. While capital, technology, and changing investment rationales present opportunities to open up previously inaccessible land, customary tenure and the lack of land markets pose major impediments for investment signalling resistance for tenurial change and commercialization. In this context, the numerous brownfield investment opportunities that are on offer for sale or lease seem to present a market solution to the many tenure-related obstacles. Such solutions have not been spontaneous, but, borne upon the legacies of previous investment failures (Kronenburg García et al., 2021).

It is also worth adding that we do not find evidence to support the previous claims that the high incidence of corruption in Africa is a key investment determinant. The investors we interviewed did not identify leniency or the tendency for corruption to be a draw, but, expressed frustration over both corruption and red tape as major costs that make investment expensive and inefficient. Our argument here assumes that the interviewees did not withhold information, which we cannot guarantee. However, within-group variability in corruption indices among the countries in Eastern and Southern Africa is low. Most investors have a regional focus, and a very few invest in land globally. This is another reason to corroborate the little if at any effect corruption may have on investment logics.

Further, our results show that the transnational investors who develop large-scale commercial agriculture and forestry in Southern and Eastern Africa are far from the homogeneous group of asset-seeking speculators, whose investments were swayed by poorly performing financial markets, high commodity prices, and attractive historical returns on farmlands (Cotula et al., 2009; Daniel, 2012; Lawrence et al., 2015). When the post-2007 land-based investment spike unfolded, farmlands in the global north were already a scarce resource and had sustained high property values (McMichael, 2012; Koeninger, 2017). Against a looming land scarcity (Lambin & Meyfroidt, 2011), investing in land is attractive for any purpose, whether rent accruing or productive purposes. While much of the scholarly focus has remained on asset-seeking institutional investors, production-oriented agribusinesses (known as the resource-seeking investors in FDI literature) have been investing in land and occupying the African frontiers for many decades. Increasingly, a single investment composes different types of investors permitting the pooling of skillsets, experiences, and capital that the investors would otherwise lack by operating independently. Therefore, we point to the importance of updating the land-based investment discourses to acknowledge the heterogeneity of investors. We also recommend the use of an investment as a more meaningful unit of observation than an investor, when assessing large-scale land investments.

Another key finding is the endogeneity of investor track records in guiding investment logics and land use. Agricultural location theory has focused on market access, production types, and agricultural suitability in explaining investment logics. Some works have found the effects of peers and neighbours to influence investment locations (Robalino & Pfaff, 2012; Garrett et al., 2013; Richards, 2018). But to the best of our knowledge, the inherent endogeneity of investor track records, i.e., the expertise they already have or have not (e.g., sector experiences, regional, experiences, and existing market reach) in guiding investment logics and land use has neither been qualified or quantified, especially within the works of land systems science. The work we present here empirically establishes how investor track records can affect land use change. For example, investors with extensive track records producing high-value crops who are motivated by ownership advantages invest preferentially in remote *subsistence frontier*. In doing so, they push the frontier, which may result in land use expansion into uncleared natural vegetation. In contrast, newcomers to a sector, including asset-seeking rentiers, focus more on *populated smallholder land* and *emerging commercial frontier*, where they seek the generic advantages of proximity to infrastructure and markets. Such knowledge can critically inform the designing and the creation of targeted investment opportunities and land use planning.

From a development point of view, we also highlight the importance of promoting and facilitating economic clustering of agricultural investments. Despite numerous investments, positive spillovers to the external economy have notably been rare in the African frontiers (Chamberlin et al., 2014; Deininger & Xia, 2016). For not-large-enough lone farmers, surviving frontiers that are below a certain minimal threshold of infrastructures and agriculture services has been difficult, even in Latin America, where agricultural markets are relatively developed (Richards, 2018). For the same reasons, state-led regional development schemes across the globe offering incentives for firms to locate in regions that lack supporting infrastructures and local expertise to leverage cluster formation, have largely been unsuccessful (Porter, 1996; Markusen & Venables, 1998; Chamberlin et al., 2014). In the case of Southern and Eastern Africa, we find a number of large-enough lone investors surviving the pre-commercial frontier, without the pre-conditions of economic clustering. These are investors who have achieved internal scale economies with large-enough patient capital. Even the much-applauded models of contract farming and out-grower schemes, managed by such large-enough lone investments, seem to have minimal effects on the broader economy (Hall et al., 2017). Given such dynamics, we propose using existing large-enough lone investments as indicators to target locating new investment clusters as opposed to scattering lone investments across the map.

Equivalent to that of deforestation in Latin America and Southeast Asia, the prevalent predicament and the dominant narrative around large-scale land investments in Africa, is the displacement of people and livelihoods. Our results point to the conditions that foster this. We find that 55% of the land across the countries we sampled to be *populated smallholder land* and 30% of the investments take place in these regions. Although these may not be the most preferred investment locations, investments that scatter across *populated smallholder land*, forestry investments in particular (33%), result in potential conflicts with local communities. Lay et al (2021) report similar results in Zambia where large-scale farms crowd-out smallholders. Spatial planning would have a key role to play in resolving such land conflicts and there are lessons that agricultural investment planning could draw from conservation planning. Based on population densities, land resources, market access, and farm concentration, land use planners can designate use and non-use zones. These delineations can range from high priority livelihood localities equivalent to no-go zones, to regions that could open up for investment under certain circumstances, to targeted investment clusters with the highest potential for economic development.

However, we wish to reiterate that issues surrounding land-based investments in Africa are wicked (Head, 2008) and any sustainable solution should be contextual and holistic. Our results clearly show the complexities involving trade-offs between conservation and sustainable rural development. For example, investors with an extensive track record continue to push the frontier. However, these same investors are more likely to survive the high transaction costs of operating in the pre-commercial frontier. Their regional and sectoral expertise and extensive market reach, indicate deep pockets and experience that allow them to weather the conditions of the pre-commercial frontiers. Compared to newcomers, their chances of stimulating employment and economic development are higher. Such trade-offs remind us why binary logics and analyses relying on normative narratives are ineffective.

We believe that these findings have important implications for investment governance, rural development, and sustainable land use planning. However, in gauging the findings, some reflection on the evolving investment context is necessary. In Africa, opposition toward large-scale commercial agriculture has been resounding and investment failure rate is high (Land Matrix, 2019; Nolte, 2020). Data from the Land Matrix (2019) and UNCTAD (2019) suggest that the number and scale of new investments are declining (Fig. S1). In parallel, attempts to improve investment governance through voluntary investment codes, obligatory funding stipulations, and the promotion of private property rights are underway (Borras Jr. & Franco, 2010; Quan & Seigneret, 2019). There has also been self-learning from the investors part. The more recent investments continue to coalesce different investors to integrate sector-specific skillsets, regional experiences, and patient-enough funding windows that were previously missing. Against this backdrop, our findings establish the need for a broader conversation around where the balance between conservation priorities, livelihoods, and rural development lies and the types of investors and investments that best contribute to achieving this. We also emphasize the importance of avoiding overly simplistic narratives.

## Supplementary information

Below is the link to the electronic supplementary material.

Supplementary file1 (PDF 1068 kb)
