# Supplementary material for: Resource frontiers and agglomeration economies: The varied logics of transnational land-based investing in Southern and Eastern Africa
Source: Ambio. 2022 Jan 15;51(6):1535–51. doi: 10.1007/s13280-021-01682-z (PMC9005594; doi:10.1007/s13280-021-01682-z)
Supplement: Supplementary file 1 — Supplementary file1 (PDF 1068 kb) [file 13280_2021_1682_MOESM1_ESM.pdf]

Ambio

Electronic Supplementary Material
This supplementary material has not been peer reviewed.

Title: **Resource frontiers and agglomeration economies: The varied logics of transnational land-based investing in Southern and Eastern Africa**

Authors: Dilini Abeygunawardane, Angela Kronenburg García, Zhanli Sun, Daniel Müller, Almeida Sitoe, and Patrick Meyfroidt

Supplementary Information (SI)

For the interview questionnaire, data, and code visit the link.

Variables: The BN included 14 predictor variables, two outcome variables, and an output typology. Table S2 lists the variables, their definitions, the states each variable take, and their thresholds.

All predictor variables were drawn from interview and additional firm data, which included 37 individual investments. The key predictor variables comprised one observed variable (type of production) and 13 latent variables, i.e., variables not directly observed, but calculated using the observed (investor track record, and a set of investment location selection criteria). The parameters for each latent predictor variable were calculated from the observed variables, outside of the net using MS Excel™ (Table S2, under 'Node states: Thresholds', explains how each variable was calculated). These observed and calculated parameters were used to populate the conditional probability tables (CPTs) of the predictor variables. CPTs take values over the interval [0, 1] (Kjærulff & Madsen, 2013).

The key outcome variable indices, i.e., *resource frontier* and *agglomeration economies*, were derived using four spatial variables. For each investment site (n=121), we derived a frontier index using spatial data on population density and the area of unconverted land and an *agglomeration economies* index using spatial data on market activity and field size (Table S3 lists data sources). We selected datasets that characterized the investment conditions as close as possible to the period when the investment decisions were made. Since BNs generally require discretizing continuous data, we used a mix of descriptive statistics and published standards to decide on the number of bins and bin-widths for the spatial variables (Table S2 lists the bins and bin-widths for spatial data). To derive the indices, we elicited a prior conditional probability for each parent state combination of the two indices, based on the definitions characterizing these indices (see Model under Methods in the main text for definitions) and our expertise in the domain (Cain, 2001). To elaborate this with an example from the *agglomeration economies* index, we take the case of an investment location that records a large field size and low-level market activity (i.e., the parent states). For this parent state combination, each of the *low*, *medium*, and *high* child states of the *agglomeration economies* index, were assigned a probability score of 0.6, 0.4, and 0, respectively. These elicited probability scores indicate that for a given investment location with a large field size and low market activity, the likelihood of the occurrence of *agglomeration economies* is more likely to be *low* with a 60 % chance, less likely to be *medium* with a 40% chance, and unlikely to be *high*. Table S4 presents the elicited conditional probabilities of the *resource frontier* index and S5 that of the *agglomeration economies* index. The conditional probability scores were arrived at by revising and deliberating the scores until there was agreement among all the authors. These outcome variable indices were then parameterized using spatial data. The probability distributions of the indices also accounted for the uncertainty in measurement errors. For parameter estimation, we used the built-in expectation-maximization (EM) algorithm in Netica™ (Lauritzen, 1995).

To facilitate interpretation, we derived an output typology by tabulating the *resource frontier* and *agglomeration economies* in a matrix. The typology was calibrated using area-weighted probability scores along the low, through medium, through high gradients of frontier and *agglomeration economies* indices (Table S6 and Fig. 1c in the main text).

Model validation: We arrived at the final BN through an iterative and interactive process involving revisions, verification, and validation (Kjærulff & Madsen, 2013). At each revision we verified the directional influence and tested the plausibility of the results. A series of final validation exercises was carried out with 3 selected investors among the sampled investors and a group of researchers who work in the region. In these final validation exercises, we asked the participants to explore the directed acyclic graph (DAG) visually and assess the conceptual framework and the plausibility of the directional influences by taking different investment

conditions as examples. We also assessed the plausibility of the overall results, against participants' expertise in the sector or in the region.

To assess the influence of each selected variable on the other variables quantitatively, we conducted a series of sensitivity tests. A sensitivity test estimates the mutual dependence between two variables, by quantifying the amount of information that an observed variable yields about another. It is an entropy reduction measure, which quantifies how much knowing one variable reduces the uncertainty regarding the other (Pearl, 1988).

SI Figures and Tables

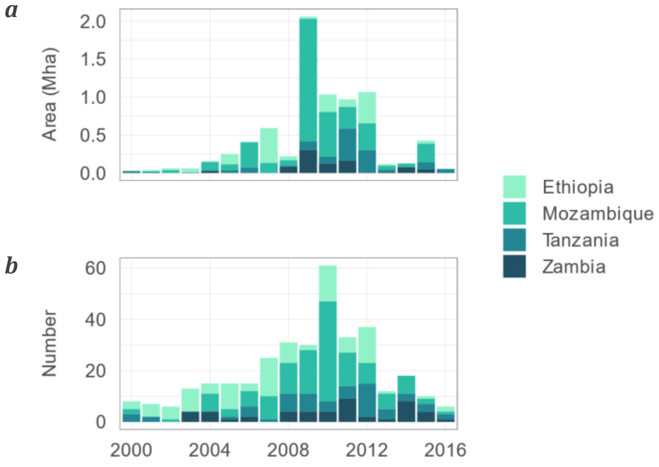

Figure S1. *Land based investment trends in Ethiopia, Mozambique, Tanzania, and Zambia between 2000 and 2016, a) area of investments in Mha and b) number of investments. Source: Land Matrix Data (2019).*

Table S1. *Economic, agriculture, and social development context in the sample countries between 2008-2011 (Source: FAOSTAT, 2019; Hansen et al., 2013; Jayne et al, 2010; World Bank, 2014, 2020).*

Country	GDP (USD billion)	Agriculture, value added (% of GDP)	Rural population (%)	Average smallholder farm size (ha)	Forested area in million ha (% of land cover)
Ethiopia	29.93	41.4	82.7	0.6	12.4 (11)
Mozambique	11.09	26.9	69.2	0.9	27.0 (34)
Tanzania	32.01	25.6	71.9	1.1	24.7 (26)
Zambia	20.27	9.4	60.6	0.9	22.4 (30)

Table S2. *Description of the investment determinants and their use in the BN. The table lists the observed variables, as described by the investors, the latent variables calculated from the observed which are included as nodes in the BN, node states, and node-state thresholds. (L) denotes a latent variable and (I) denotes an index.*

**BN nodes**	**Observed variables**	**Node states: Thresholds**
**Predictor variables: Investor profile**		
1. Investor track record (L)	Aggregate effect of product market reach, skillset, and regional experience	*none*: all the child/observable variables in none state,<br>*limited*: one child variable is in none state or both child variables in limited state,<br>*extensive*: at least one child variable is in extensive state and others in limited or extensive state
a. Product market reach (L)	Degree of local and export market reach	*none*: no market reach,<br>*limited*: only local market reach,<br>*extensive*: at least some export market reach
i. Export market reach (L)	Export market for the invested crop is already established	*no*: no established export product market,<br>*yes*: export product market already established
ii. Local market reach (L)	Local market for the invested crop is already established	*no*: no established local product market,<br>*yes*: local product market already established
b. Skillset (L)	Aggregate effect of farming or forestry experience in the same or similar types of production and other farming or forestry experience	*none*: all the child variables in no state,<br>*limited*: all the child variable combinations except none and extensive states,<br>*extensive*: child variables farming or forestry experience in the same or similar types of production AND regional experience are in yes state
i. Farming or forestry experience in similar types of production (L)	Previous farming or forestry experience in the same or similar types of production as the target production of the investment under study	*no*: no farming or forestry experience in the same or similar types of production,<br>*yes*: has farming or forestry experience in the same or similar types of production
ii. Farming or forestry experience in other types of production (L)	Previous farming or forestry experience in types of production other than the target production of the investment under study	*no*: no farming or forestry experience,<br>*yes*: has farming or forestry experience in other types of production
iii. Regional experience (L)	Previous experience in farming, forestry, trading, or in any commercial activity in Southern and Eastern Africa	*no*: no commercial experience in the region,<br>*yes*: previous commercial experience in the region in farming, forestry, trading, or any other commercial activity
2. Types of production	Type of production	*high-value food crops*: high-value deciduous fruits and nuts,<br>*forestry*: logging and plantation forestry,<br>*other*: other agriculture excluding high value food crops
**Predictor variables: Investment selection criteria (aggregate effect of the priorities assigned by the investor to the observable variables);**		
1. Agroecology (L)<br><br>*Note: Frequency of agroecology outside of the BN measures at least one positive observed variable in the group*	Irrigation, Altitude, Soils, Other agroecological conditions	*low*: less than two of the child variables are in high sate,<br>*high*: at least two of the child variables are in high state
2. Infrastructure and logistics (L)	Access to utility services, Means of transport, Access to processing and storage	*low*: all the child variables are in low state,<br>*high*: at least one child variable is in high state
a. Means of transport (L)	Transport logistics, Infrastructure	"
3. Land accessibility (L)	Extent of land, Land conflicts, Population density, Land price, Brownfields	"
4. Market proximity (L)	Proximity to local markets and distribution hubs, Proximity to international markets	"
5. Labour supply (L)	Labour skillset, Labour availability, Cost of labour	"
6. Policy environment (L)	Agriculture policy, Trade policy, Land policy, Investment policy	"
7. Strategic reasons (L)	Vertical integration, Competitive advantage, Diversification, Upstream-and downstream balance	"
8. Economic and financial conditions (L)	The proportion of agricultural population to GDP, Long-term investment, Cashflow, Availability of money	"
9. Operations-related reasons (L)	Operational cost, Strong local partners, In situ experience, Technical capacity	"
10. Socio-economic and environmental impact (L)	Environmental impact, Socio-economic impact	"
11. Market drivers (L)	Product price, Demand, Traditional growing areas, Guaranteed market, Timing of the markets	"
12. Other inputs supply (L)	Input supplies, GMOs, Access to forestry(wood), Mechanization	"
Governance	Political stability, Bureaucracy and red tape, Rule of law	Excluded from the BN due to the very low frequency in citing
Pioneering spirit	Pioneering spirit	Excluded from the BN due to the very low frequency in citing

**Outcome variables**		
1. *Resource frontier* (I)	Calibrated based on population density and unconverted land	*low*, *medium*, and *high*: See Table S4 for thresholds
a. Population density	People per square kilometre	*low*: <25/km², *medium*: 25-50/km², *high*: >50/km²
b. Unconverted land	Proportion of land unconverted to agriculture but potentially suitable (excluding deserts and bare lands)	*low*: <10%, *medium*: 10-20%, *high*: >20%
2. *Agglomeration economies* (I)	Calibrated based on field size and market activity	*low*, *medium*, and *high*: See Table S5 for thresholds
a. Field size	Enclosed agricultural areas, including annual and perennial crops	*low*: <0.64 ha, *medium*: 0.64 -16ha, *high*: >16ha
b. Market activity	Travel time to influential markets	*low*: <0.1, *medium*: 0.1-0.3, *high*: >0.3
**Output typology**		
Investment location (I)	Typology based on *resource frontier* and *agglomeration economies* indices	*populated smallholder land*, *subsistence frontier, emerging commercial frontier*, and *established market*: See Table S6 and Fig. 1c in the main text for thresholds

Table S3. *Descriptions and sources of spatial data.*

Variable	Years of data sets	Spatial resolution (km^2)	Description	Source
Land cover	2010	0.3 x 0.3	Continuous land cover changes at 300 m resolution; land cover classes reclassified as unconverted land includes gird cells with; > 50% of mosaic shrub herbaceous cover, > 15% of broadleaved evergreen canopy cover, > 15% of broad leaved deciduous closed to open tree cover, > 50% mosaic tree and shrub or < 50% herbaceous cover, > 50% mosaic herbaceous cover, shrubland, and grassland	Li et al. (2018)
Population density	2000	0.6 x 0.6	Mosaiced 1km resolution global data sets using sub-national census-based population estimates interpolated at 100m using Random Forest estimation	Lloyd et al. (2019); WorldPop and CIESIN (2018)
Field size	2017	0.9 x 0.9	Enclosed agricultural areas, including annual and perennial crops mapped at 30m resolution	Lesiv et al. (2019)
Market influence	1979-2010	8.4 x 8.4	Travel time accounting for infrastructure and terrain to influential markets weighted by GDP PPP	Verburg, Ellis, and Letourneau (2011)

Table S4. *Elicited probability table used in calibrating the priors for the resource frontier index. The values represent an expert derived probability score of how likely the different states of the child node (i.e., the resource frontier variable) are, given the different states of the two parent nodes (i.e., population density and unconverted land). For e.g., the first line of the table reads, if population density is low and unconverted land is low, there is a 60 % chance that resource frontier is low, a 40 % chance that it is medium, and no chance that it is high.*

Population density	Unconverted land	***Resource Frontier***		
		**low**	**medium**	**high**
low	low	0.6	0.4	0
low	medium	0	0.6	0.4
low	high	0	0	1
medium	low	0.8	0.2	0
medium	medium	0	0.8	0.2
medium	high	0	0.4	0.6
high	low	1	0	0
high	medium	0.2	0.8	0
high	high	0.1	0.6	0.3

Table S5. *Elicited probability table used in calibrating the priors for the agglomeration economies index. The values represent an expert derived probability score of how likely the different states of the child node (i.e., the agglomeration economies variable) are, given the different states of the two parent nodes (i.e., field size and market activity). For e.g., the first line of the table reads, if field size is large and market activity is low, there is a 60 % chance that agglomeration economy is low, 40 % chance that it is medium, and no chance that it is high.*

**Field size**	**Market activity**	***Agglomeration economies***		
		**low**	**medium**	**high**
large	low	0.6	0.4	0
large	medium	0	0.4	0.6
large	high	0	0	1
medium	low	0.8	0.2	0
medium	medium	0	0.6	0.4
medium	high	0	0.2	0.8
small	low	1	0	0
small	medium	0.4	0.6	0
small	high	0.3	0.6	0.1

Table S6. *Elicited probability table used in calibrating investment location typology. The values represent a probability score weighted by area to classify how likely the different investment locations are, along the low, through medium, through high gradients of resource frontier and agglomeration indices. See Fig. 1c in the main text for a graphical representation of the scores.*

Resource frontier	***Agglomeration economies***	**Investment location**			
		Populated smallholder land	***Subsistence frontier***	***Emerging commercial frontier***	***Established market***
low	low	1	0	0	0
low	medium	0.2	0	0	0.8
low	high	0	0	0	1
medium	low	0.2	0.8	0	0
medium	medium	0.04	0.16	0.64	0.16
medium	high	0	0	0.8	0.2
high	low	0	1	0	0
high	medium	0	0.2	0.8	0
high	high	0	0	1	0

SI References

Cain, J. (2001). *Planning Improvements in Natural Resources Management: Guidelines for using Bayesian networks to support the planning and management of development programmes in the water sector and beyond* (Vol. 124). Wallingford, UK: Centre for Ecology and Hydrology.

FAOSTAT. (2019). Retrieved January 16 2019, from http://www.fao.org/faostat/

Hansen, M. C., Potapov, P. V., Moore, R., Hancher, M., Turubanova, S. A., Tyukavina, A., . . . Townshend, J. R. G. (2013). High-Resolution Global Maps of 21st-Century Forest Cover Change. *Science 342*, 850–853. Retrieved from https://glad.umd.edu/dataset/global-2010-tree-cover-30-m

Jayne, T. S., Mather, D., & Mghenyi, E. (2010). Principal Challenges Confronting Smallholder Agriculture in Sub-Saharan Africa. *World Development, 38*(10), 1384-1398. doi:10.1016/j.worlddev.2010.06.002

Kjærulff, U. B., & Madsen, A. L. (2013). *Bayesian Networks and Influence Diagrams: A Guide to Construction and Analysis*. New York, NY: Springer New York.

Land Matrix. (2019). Retrieved 15 July 2019, from https://landmatrix.org/

Lauritzen, S. L. (1995). The EM algorithm for graphical association models with missing data. *Computational Statistics & Data Analysis, 19*(2), 191-201. doi:10.1016/0167-9473(93)E0056-A

Lesiv, M., Laso Bayas, J. C., See, L., Duerauer, M., Dahlia, D., Durando, N., . . . Fritz, S. (2019). Estimating the global distribution of field size using crowdsourcing. *Global Change Biology, 25*(1), 174-186. doi:doi:10.1111/gcb.14492

Li, W., MacBean, N., Ciais, P., Defourny, P., Lamarche, C., Bontemps, S., . . . Peng, S. (2018). Gross and net land cover changes in the main plant functional types derived from the annual ESA CCI land cover maps (1992–2015). *Earth Syst. Sci. Data, 10*(1), 219-234. doi:10.5194/essd-10-219-2018

Lloyd, C. T., Chamberlain, H., Kerr, D., Yetman, G., Pistolesi, L., Stevens, F. R., . . . Tatem, A. J. (2019). Global spatio-temporally harmonised datasets for producing high-resolution gridded population distribution datasets. *Big Earth Data, 3*(2), 108-139. doi:10.1080/20964471.2019.1625151

Pearl, J. (1988). *Probabilistic Reasoning in Intelligent Systems: Networks of Plausible Inference*: Morgan Kaufmann Publishers Inc.

Verburg, P. H., Ellis, E. C., & Letourneau, A. (2011). A global assessment of market accessibility and market influence for global environmental change studies. *Environmental Research Letters, 6*(3), 034019. doi:10.1088/1748-9326/6/3/034019

World Bank. (2014). *Enquête Agricole de Conjoncture Intégrée 2014 (EAC-I 2014)*. Retrieved from https://microdata.worldbank.org/index.php/catalog/2583

World Bank. (2020). *World Development Indicators*. Retrieved from https://databank.worldbank.org/source/world-development-indicators

WorldPop, & CIESIN. (2018). *Global High Resolution Population Denominators Project.* Retrieved from www.worldpop.org
